# Institutionalizing a Regional Model for Improving Quality of Newborn Care at Birth Across Hospitals in Eastern Uganda: A 4-Year Story

**DOI:** 10.9745/GHSP-D-20-00156

**Published:** 2021-06-30

**Authors:** Peter Waiswa, Phillip Wanduru, Monica Okuga, Darius Kajjo, Doris Kwesiga, James Kalungi, Harriet Nambuya, Jude Mulowooza, Abner Tagoola, Stefan Peterson

**Affiliations:** a Makerere University School of Public Health, Kampala, Uganda.; b Global Public Health, Karolinska Institute, Stockholm, Sweden.; c Busoga Health Forum, Jinja, Uganda.; d Uppsala University, Uppsala, Sweden.; e Jinja Regional Referral Hospital, Jinja, Uganda.; f Iganga General Hospital, Iganga, Uganda.

## Abstract

**Introduction:**

Despite the rapid increase in facility deliveries in Uganda, the number of adverse birth outcomes (e.g., neonatal and maternal deaths) has remained high. We aimed to codesign and co-implement a locally designed package of interventions to improve the quality of care in hospitals in the Busoga region.

**Design and Implementation:**

This project was designed and implemented in 3 phases in the 6 main hospitals in east-central Uganda from 2013 to 2016. First, the inception phase engaged health system managers to codesign the intervention. Second, the implementation phase involved training health providers, strengthening the data information system, and providing catalytic equipment and medicines to establish newborn care units (NCUs) within the existing infrastructure. Third, the hospital collaborative phase focused on clinical mentorship, maternal and perinatal death reviews (MPDRs), and collaborative learning sessions.

**Achievements:**

In all 6 participating hospitals, we achieved institutionalization of NCUs in maternity units by establishing kangaroo mother care areas, resuscitation corners, and routine MPDRs. These improvements were associated with reduced maternal and neonatal deaths. Facilitators of success included a simple, low-cost, and integrated package designed with local health managers; the emergence of local neonatal care champions; implementation and support over a reasonably long period; decentralization of newborn care services; and use of mainly existing local resources (e.g., physical space, human resources, and commodities). Barriers to success related to limited hospital resources, unstable electricity, and limited participation from doctors. More advanced NCUs have been established in 3 of the 6 hospitals, and 7 high-volume comprehensive health centers have been established with functional NCUs.

**Conclusion:**

The involvement of local health workers and leaders was the foundation for designing, sustaining, and scaling up feasible interventions by harnessing available resources. These findings are relevant for the quality of care improvement efforts in Uganda and other resource-restrained settings.

## Abstract (precis)

A locally developed, low-cost package of interventions implemented in a regional network of hospitals resulted in significant reductions in mortality for mothers and newborns as well as the institutionalization of the quality improvement initiative. This work demonstrates that it is possible to achieve the World Health Organization/United Nations Children's Fund Quality of Care targets in hospitals.

## Teaser

**Key Findings:**

- The interventions implemented led to improvements in maternal and newborn outcomes and the institutionalization and scale-up of the quality improvement process at both hospitals and high-volume health centers.
- Critical factors for success included codesigning and implementing a package of services that fit the context. This package was locally led, integrated but simple, and built on and sustained by mainly local resources to develop local champions.
- Challenges included lack of participation from doctors due to high doctor-patient ratio, scarcity of human and other resources, inadequate infrastructure, unstable electricity, and multiplicity of other initiatives.

**Key Implications:**

- Successful quality improvement projects must be integrated but feasible, based on the context and building onto the available resources, and implemented over a fairly long time. Creating a network of regional hospitals via a quality improvement collaborative rapidly leads to improved referral care and increased access to high-quality services for mothers and newborns. Once these efforts are operationalized and functional in hospitals, high-volume primary health centers can be brought aboard, which leads to scale-up.
- Having a network of hospitals working to improve quality can be a platform for learning, research, and knowledge generation and for building advanced neonatal care units in places where they hitherto never existed. However, such efforts must be complemented by recruiting more specialized health workers, such as neonatal nurses, pediatricians, and neonatologists, into regional and district hospitals so that nurses and midwives are well supported.

## BACKGROUND

In 2015, the global policy architecture transitioned from Millennium Development Goals to Sustainable Development Goals, which aim to reduce global maternal mortality by two-thirds, or 70/100,000 live births, by 2030, with no country exceeding a maternal mortality rate of 140/100,000 live births.^1^ Similarly, the target newborn mortality rate was set at fewer than 12/1,000 live births by 2030.^2^ Whereas many high- and middle-income countries have made significant milestones toward these targets, major challenges remain in most low-income countries.

In Uganda, a low-income country, the maternal mortality ratio is estimated at 375/100,000 live births, which is an improvement from 438/100,000 live births in 2011,^3^^,^^4^ but it is still far from the Sustainable Development Goals target. In addition, the country's newborn mortality has stagnated at 27/1,000 live births for over a decade.^3^^,^^4^ Despite nearly three-quarters of births occurring in health facilities, Uganda's maternal and newborn mortality rates remain high, which reflects the limited quality of care during pregnancy, labor, and postnatal periods. The consequences of these gaps in care include an estimated 6,000 maternal deaths, 32,300 neonatal deaths, and 34,150 stillbirths per year in the country.^5^ Most of these deaths can be prevented through the provision of high-quality care around the time of birth.^6^^–^^8^

Uganda's maternal and newborn mortality rates remain high, despite nearly three-quarters of births occurring in health facilities.

In response to the need to accelerate reductions in mortality, the World Health Organization (WHO) initiated the Quality of Care Network aimed at reducing maternal, neonatal, and child mortality in institutions by implementing a set of standards.^9^ Although Uganda's Ministry of Health (MOH) focuses on improving facility care around the time of birth, as evidenced in its policy documents,^10^ there is limited understanding of how this can be achieved in a scalable manner using available resources. A few large-scale maternal and newborn projects have been implemented successfully, such as the Saving Mothers Giving Life project, but sustainability remains a challenge.^11^ Key contributing factors to the lack of sustainability include high resource intensity and too many simultaneous interventions overwhelming the local health system.^11^^,^^12^

Considering sustainability challenges that have hindered past large-scale maternal and newborn project interventions, we designed and implemented a low-cost, limited-intensity package to improve care at birth. This report describes the implementation experiences and lessons learned to inform maternal and newborn health policy formulation and implementation of similar initiatives in Uganda and elsewhere. The findings presented in this report are based on a synthesis of quarterly project reports and routine health information system data, which was conducted prospectively. This report details activities, challenges, and lessons learned, as well as changes in select indicators (e.g., mortality, delivery, and admissions).

Considering sustainability challenges that have hindered past large-scale maternal and newborn project interventions, we designed and implemented a low-cost, limited-intensity package to improve care at birth.

### Implementation Setting

**FIGURE 1:**
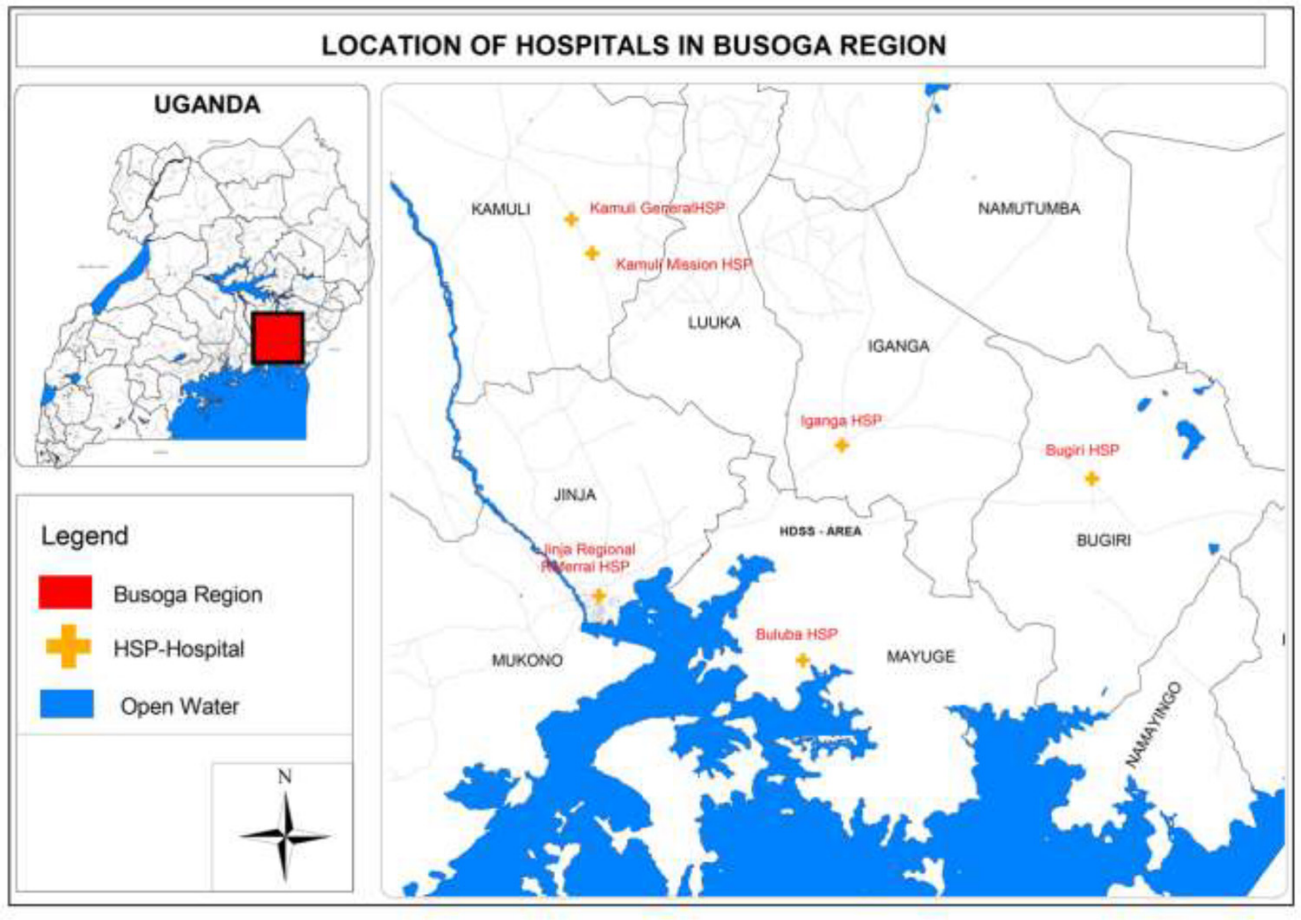
Location of Hospitals Where a Locally Designed Package of Interventions to Improve Quality of Maternal and Newborn Care Was Implemented, Busoga Region, Uganda

This package was implemented between 2013 and 2016 in 6 hospitals that collaborated to improve the quality of care at birth in their catchment area, a region of about 4 million people. The hospitals included 1 regional referral hospital and 5 general hospitals (including 2 private not-for-profit missionary hospitals) (Figure 1). All hospitals provide several services (e.g., general preventive and promotional, outpatient and inpatient curative, maternity and obstetric, pediatric, emergency and specialist surgery, blood transfusion, and laboratory services). The regional referral hospital employs consultant pediatricians, surgeons, physicians, and obstetricians and thus provides highly specialized services not available at other facilities. The regional referral hospital also is mandated with supervising and building the capacity of district hospitals within the region. This region has a high fertility rate of 6.1.^3^ On average, these 6 hospitals conduct about 1,800 deliveries per month (Table 1).

**TABLE 1.** Description of Hospitals and Their Maternal and Newborn Care Capacity Where a Locally Designed Package of Interventions to Improve Quality of Maternal and Newborn Care Was Implemented, Busoga Region, Uganda

	Level, Ownership	Cadres Available, No.	Mean Monthly Deliveries
Jinja Hospital	Regional referral hospital, public	Consultant pediatricians, 1 Obstetricians, 3 Pediatricians, 2 Medical officers, 4–7 Midwives, 13–20 Neonatal nurses, 1 Anesthetic officers, 3–7	500
Iganga Hospital	General hospital, public	Obstetrician, 1 Medical officers, 11 Midwives, 12–15 Anesthetic officers, 1–3	550
Bugiri Hospital	General hospital, public	Medical officers, 2–3 Midwives, 8–12 Anesthetic officers, 1–3	250
Kamuli General Hospital	General hospital, public	Medical officers, 5–7 Midwives, 15–20 Anesthetic officers, 1–3	200
Kamuli Mission Hospital	General hospital, private not-for-profit	Medical officers, 3 Midwives, 7–10 Anesthetic officers, 1–3	200
St. Francis Buluba General Hospital	General hospital, private not-for-profit	Medical officers, 4 Midwives, 7–10 Anesthetic officers, 1–3	100
			1,800 total

## THE INTERVENTION

**FIGURE 2:**
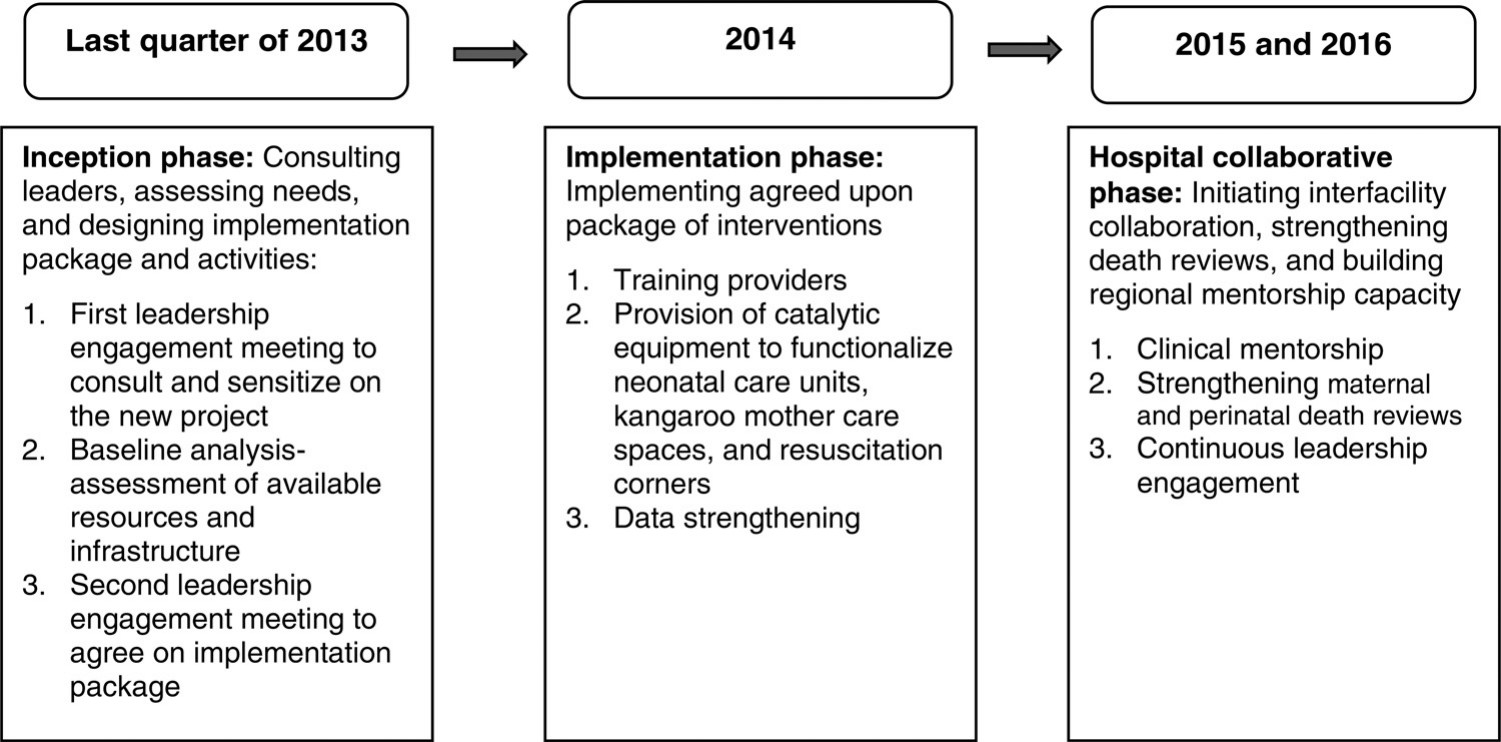
Schematic Representation of the Project Implementation to Improve Quality of Maternal and Newborn Care in Hospitals, Busoga Region, Uganda

The package was designed and implemented through a collaboration between health managers and project staff. The aim was to improve the quality of care at birth using mostly locally available human resources, commodities, supplies, and infrastructure. The intervention was designed and implemented in 3 major phases: inception, the implementation phase, and hospital collaborative (Figure 2).

### Phase 1: The Inception Phase

#### Engage Health System Managers

Phase 1 took place from November to December 2013. It comprised an initial 1-day health system managers' engagement meeting, followed by a baseline health facility readiness survey and a second engagement meeting. The initial health system managers' meeting was attended by 6 medical superintendents, 6 hospital administrators, and 6 maternity unit nurses in-charge from the 6 hospitals; 5 district health officers; 2 MOH obstetricians; and 2 pediatricians from the 5 districts in which the hospitals are located. This engagement meeting was aimed at discussing the new project, the proposed approach, the manager's anticipated roles, and the forthcoming baseline survey at their facilities. Meeting attendees provided input on the intervention package and suggestions for how to run the project. In addition, we sought their cooperation in granting research teams access to data and responding openly, especially about their challenges and suggested solutions.

#### Conduct Baseline Health Facility Assessment

The baseline survey included a health facility readiness assessment using WHO's service availability and readiness assessment tool.^13^ Semi-structured interviews were conducted with health providers and facility managers. Key findings from a baseline survey showed a lack of infrastructure for newborn care in the maternity units, limited provider competence in managing newborns, lack of drugs for managing newborn complications, and poor data quality in terms of completeness and accuracy. Opportunities for quick improvement were identified, including the availability of space that could be used to establish newborn care units (NCUs) within maternity units, availability of an existing NCU at the regional hospital from which other hospitals could learn, and willingness of leaders from the regional referral hospital to support the process of improving newborn care at other hospitals.

Baseline survey findings showed a lack of infrastructure for newborn care in the maternity units, limited provider competence in managing newborns, lack of drugs for managing newborn complications, and poor data quality in terms of completeness and accuracy.

#### Cocreate Intervention

The second health system managers' engagement meeting was a cocreation meeting involving dissemination of findings from the baseline survey and discussions with the leaders geared toward identifying priority interventions for implementation to address key findings. This meeting was facilitated by the principal investigator of the study (PW). The general guidelines for the package to be developed were that it had to be simple, low in cost, and built around the available project and hospital resources. Participants of this cocreation meeting agreed on an initial package of interventions targeted at improving capacity for providers to care for mothers and their newborns while concurrently addressing key health system gaps to facilitate newborn care, including functionalizing NCUs and improving the quality of routine data.

### Phase 2: The Implementation Phase

In this phase, we implemented the interventions agreed upon at the cocreation meeting through 3 main activities: training frontline health workers, establishing functional NCUs; and strengthening the data system. This phase lasted for 1 year (all of 2014).

#### Train Health Workers

An integrated basic emergency obstetric and newborn care didactic training began in January 2014. We used a 5-day training module, adopted from the MOH training package, to train 90 nurse-midwives in 5 separate training groups. All training sessions took place at the regional referral hospital and were facilitated by national and local trainers, including obstetricians, pediatricians, and midwives.

#### Establish NCU and Procure Drugs and Commodities

Next, the kangaroo mother care rooms, resuscitation corners, and NCUs were set up in the hospitals by working with hospital managers to identify appropriate spaces within the maternity units. We procured essential catalytic newborn care drugs, supplies, and equipment to ensure the functionalization of these units. The commodities included drugs (e.g., antibiotics, anticonvulsants, analgesics, fluids, and bronchodilators), equipment (e.g., newborn feeding equipment, resuscitation equipment, incubators, cribs, phototherapy machines, radiant warmers, and oxygen concentrators), and chairs to aid with kangaroo mother care. The trained health care providers gradually started managing sick newborns in these established spaces. Because hospital leaders were part of the initiative, they took over the sustainability of commodities by replenishing drugs and allocating staff to the NCUs.

#### Strengthen Data Systems

Furthermore, we conducted data systems strengthening to improve record quality. Data strengthening activities included developing and providing previously nonexistent national-level registers of sick newborns for the NCUs. We also provided refresher training sessions on how to enter data into the newborn and maternity registers and how to use data for local improvement activities. These 2 registers are the primary data collection tools and thus directly affect the data quality reported in the DHIS2 system that in turn feeds into the MOH data system. These registers also served as the data source for the project evaluation.

### Phase 3: Hospital Collaborative Phase

Between 2015 and 2016, we started a hospital collaborative to promote peer learning. Across hospitals, we also intensified additional follow-up on activities to include on-the-job mentorships, maternal and perinatal death reviews (MPDRs), and learning sessions, as detailed below.

#### Establish On-the-job Clinical Mentorship

On-the-job clinical mentorships involved practical coaching and consultation between a mentor (a more experienced clinician) and a mentee (a qualified but less experienced health worker). The overall purpose was to promote good clinical practices and subsequently improve the quality of care provided. The selection of 16 mentors was based on their positions, academic qualifications, years of experience, and willingness to be champions of change. We first selected 3 obstetricians, 2 pediatricians, and 3 midwives from within the region who were experienced and already working as local champions. We then added 8 more mentors (3 pediatricians, 3 obstetricians, 1 neonatal nurse, and 1 midwife) from the national level who had been engaged in various mentorship programs. An adequate number of mentors is critical to ensure a minimum number (e.g., 3) is always available for field work.

The mentorship activity was implemented in 2 phases. In the first stage, which lasted 6 months, we paired a local mentor with a national-level mentor at each facility. Pairing local mentors with national-level mentors helped build local mentor capacity and ensure long-term sustainability. In the second stage, which lasted until the end of 2016, we gradually stopped using the national-level mentors until only 3 remained. During this stage, local mentors acquired the skills to serve as mentors to the health workers in all the hospitals. We also were confident in the capacity of the local mentors, based on observations and feedback from national-level mentors.

Pairing local mentors with national-level mentors helped build local mentor capacity and ensure long-term sustainability.

The mentorship implementation format involved mentors spending 2 days per month at each hospital, during which they worked with primary providers engaged in a range of activities, including clinical rounds, delivery, and resuscitation of newborns. In the process, mentors taught and demonstrated good clinical management practices to the mentees. In the first 4 months of mentorship, mentors worked with mentees on any case at the facility without being selective.

After 4 rounds of mentorship, the local health providers expressed concerns about mentors spending much of the time coaching them on how to manage cases that they already knew how to handle, and thus not much value was added. In response, we linked MPDRs to the mentorship to identify system failures that led to a maternal or perinatal death. The identified failures were then used to generate change ideas, which were addressed during the clinical mentorship.

Mentorship implementation was guided by the plan-do-study-act approach.^14^ Using recommendations from the MPDRs, the mentors worked with health workers to develop and test ideas that they believed would improve the situation. Table 2 shows some of the most successful changes. For changes that required capacity building or changes to clinical management procedures, the mentors worked with providers to model the proper practices. These change ideas were implemented and followed up during the next mentorship visit. The lessons from implementing these change ideas were disseminated during learning sessions with other facilities.

**TABLE 2.** Examples of Successful Change Ideas Generated by Mentors and Mentees for Addressing System Failures That Lead to Maternal or Perinatal Deaths, Busoga Region, Uganda

Category	Idea	Where It Worked Well
Supplies	Keep a buffer stock of essential supplies that typically run out (e.g., blood products, medicines for managing pre-eclampsia and eclampsia, anticonvulsants for newborns, intravenous antibiotics, sterile gloves)	All public facilities
	Work with district health office leadership to access excess supplies, such as drugs, from less busy lower-level facilities	All public facilities
Clinical care	Establish a triage area and triage checklist to ensure all mothers are screened at admission to identify critical cases	All facilities (public and PNFP)
	Use a predischarge checklist in the postnatal unit to ensure all mothers and babies who are discharged are stable	All facilities (public and PNFP)
	Arrange the maternity unit so that unstable mothers requiring constant monitoring are close to nurse workstations	All facilities (public and PNFP)
	Ensure every mother is reviewed by a medical doctor or obstetrician before delivery	1 public and 1 PNFP
	Ensure 80% partograph use for all mothers in labor	All facilities (public and PNFP)
	Assess blood pressure of all mothers at least twice before delivery (within 1 hour of birth and before discharge)	All facilities (public and PNFP)
	Ensure all mothers who enter the NCU wear a gown over their clothes to prevent infection	At facilities with access to washing machine
	Place hand sanitizer at all NCU entrances to prevent infection	All facilities (public and PNFP)
Administrative	Ensure representation of hospital administrator in maternity department meetings, including routine and non-routine (e.g., maternal and perinatal death review) meetings	All facilities (public and PNFP)
Data-related	Ensure all deliveries are entered in the maternity register and all NCU admissions in the newborn register	All facilities (public and PNFP)

Abbreviations: NCU, newborn care unit; PNFP, private not-for-profit.

#### Implement Maternal and Perinatal Death Reviews

At the beginning of the project, the team intended to implement MPDRs as a stand-alone intervention. However, MPDRs eventually were linked with mentorship, as previously described. In the first 3 months of the hospital collaborative phase, the mentors conducted clinical mentorship and supported facilities in initiating the implementation of regular death reviews.

Before starting this implementation, the project team engaged the hospital and maternity unit managers to form hospital MPDR committees and to schedule meeting days. The project provided some funding to procure refreshments during these meetings. The mentors supported the capacity building of the providers to conduct proper death reviews, presided over the initial MPDR meetings, and encouraged a blame-free death review environment so that teams could generate specific, measurable, attainable, realistic, and time-bound actionable recommendations. By the third month, teams were able to work independently and had appointed chairpersons (usually the maternity unit nurse in-charge) for the meetings. The mentors then assumed a more passive and supportive role, reminding teams to review the deaths.

#### Conduct Collaborative Learning Sessions

As previously described, the implementation of clinical mentorship and MPDRs using a plan-do-study-act framework resulted in identifying and testing change ideas. Every quarter, we conducted 7 collaborative learning sessions with health system managers from all 6 hospitals to share lessons learned from implementing change ideas. The team from each facility, led by the maternity nurse in charge, highlighted which solutions worked and why others failed. Colleagues from other facilities asked questions with the intention to learn and possibly transfer best practices to their setting.

Facilities also presented data on selected performance indicators (e.g., partograph use and proportion of babies in NCU who were discharged alive). They then explained any changes in the indicators, highlighting major accomplishments and barriers.

## ACHIEVEMENTS OF THE INTERVENTION

As shown in Table 3, at the beginning of the project in 2013, only 1 facility had a functional NCU in the maternity unit. By the end of 2016, all facilities had functional NCUs, kangaroo mother care rooms, and resuscitation corners in their maternity units. Although initiated with support from the project, hospital management eventually took on the role of sustaining these units. Hospital managers and mentors became champions of newborn care at their facilities, ensuring these services were sustained.

**TABLE 3.** Achievement of Interventions to Improve Quality of Maternal and Newborn Care Across 6 Hospitals in Busoga Region, Uganda

Indicators Monitored	2013	2014	2015	2016
**Inputs, no. (%)**				
Hospitals with designated resuscitation corner	2 (33)	6 (100)	6 (100)	6 (100)
Hospitals with working resuscitation corner	2 (33)	6 (100)	6 (100)	6 (100)
Hospitals with designated space for the KMC space	2 (33)	6 (100)	6 (100)	6 (100)
Hospitals with KMC room and mothers practicing KMC	1 (17)	5 (83)	5 (83)	5 (83)
Hospitals with designated NCU space in maternity unit	3 (50)	6 (100)	6 (100)	6 (100)
Hospitals with functioning NCU admitting and managing babies in the maternity unit	1 (17)	6 (100)	6 (100)	6 (100)
**Processes**				
Staff receiving training, no.	0	90	0	0
Hospitals receiving mentorship, no. (%)	0 (0)	0 (0)	6 (100)	6 (100)
Mentoring visits per facility, no.	0	0	6	6
Hospitals conducting maternal audits, no. (%)	2 (33)	2 (33)	6 (100)	6 (100)
Maternal deaths audited, no.	18	12	45	49
Hospitals conducting weekly perinatal audits, no. (%)	0 (0)	0 (0)	6 (100)	6 (100)
Perinatal deaths audited, no.	0	0	35	36
**Outputs, no.**				
Deliveries	19,457	20,338	20,905	21,681
NCU admissions	800	1,645	2,661	3,805
Cesarean deliveries	4,807	4,838	5,724	5,595

Abbreviations: KMC, Kangaroo Mother Care; NCU, newborn care unit.

In terms of capacity building, 90 frontline nurses and midwives at 6 hospitals were trained in the management of maternal and newborn health complications. Furthermore, in 2013 and 2014, only 2 facilities conducted death audits, and they were not regular. In response to the MPDR strengthening effort, all facilities started conducting regular MPDRs.

The sick newborn register that was introduced during the data strengthening period was adopted by the MOH and is now used nationwide.

**FIGURE 3:**
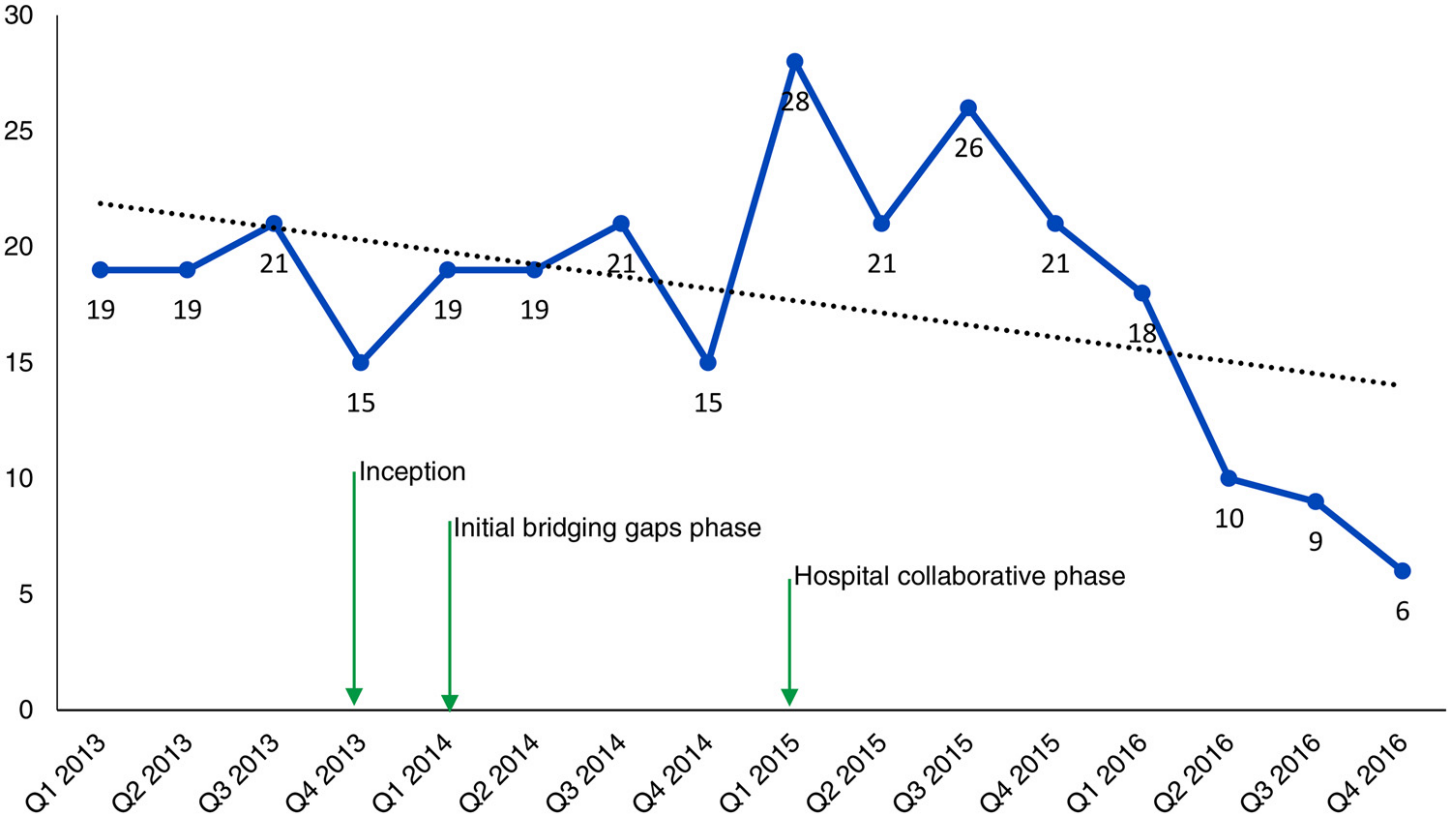
Changes in Number of Maternal Deaths in Hospitals Where a Locally Designed Package of Interventions to Improve Quality of Maternal and Newborn Care Was Implemented, Busoga Region, Uganda

**FIGURE 4:**
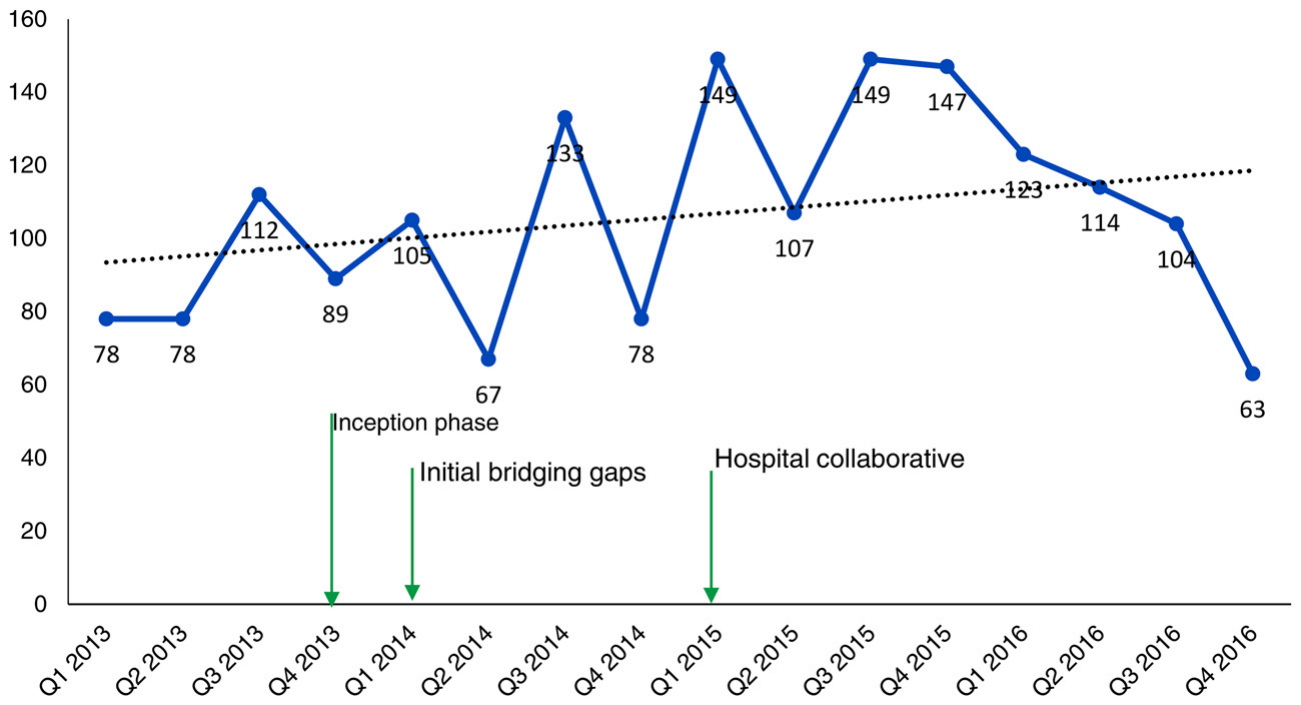
Annotated Chart for Predischarge Neonatal Mortality Rate in Hospitals Where a Locally Designed Package of Interventions to Improve Quality of Maternal and Newborn Care Was Implemented, Busoga Region, Uganda

**FIGURE 5:**
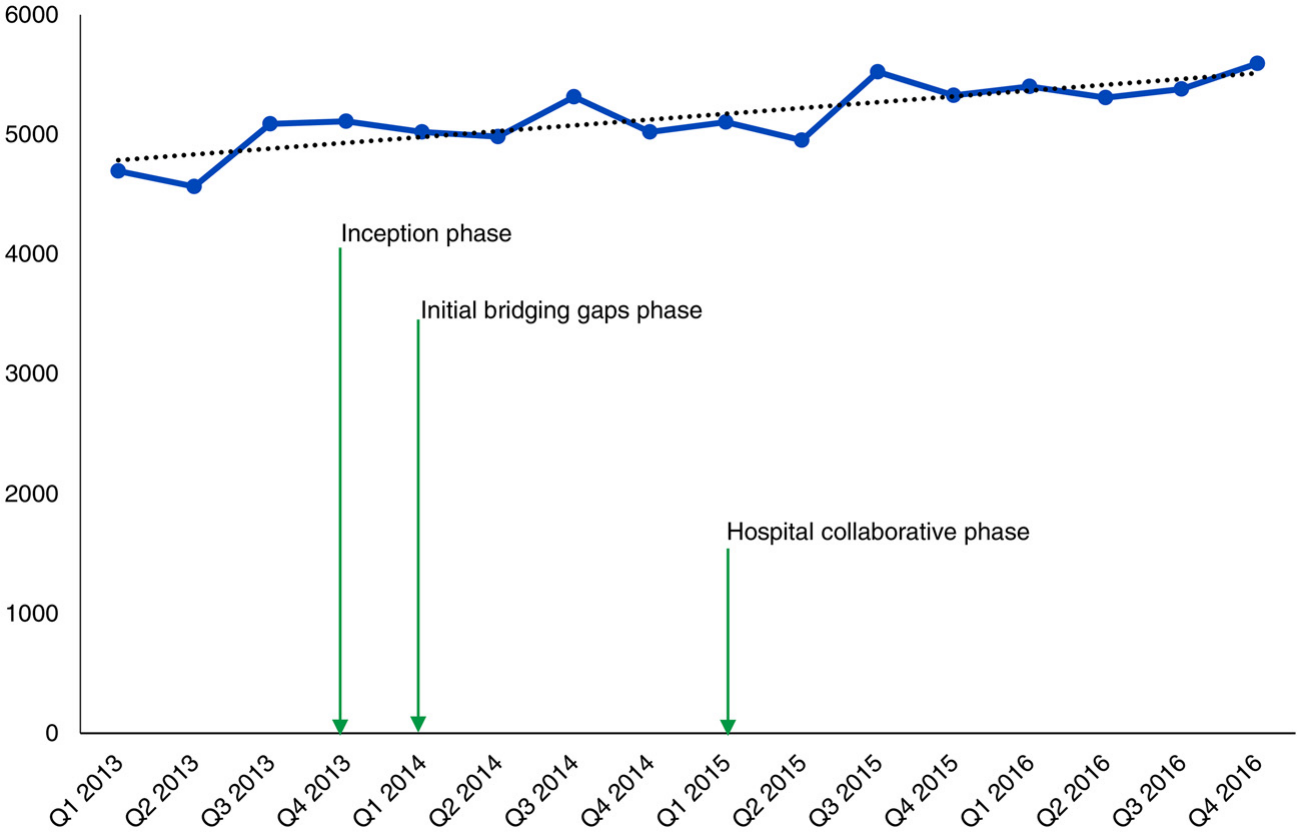
Trends in Deliveries Over Time With Initiation of Implementation Phases of Project to Improve Quality of Maternal and Newborn Care in Hospitals, Busoga Region, Uganda

**FIGURE 6:**
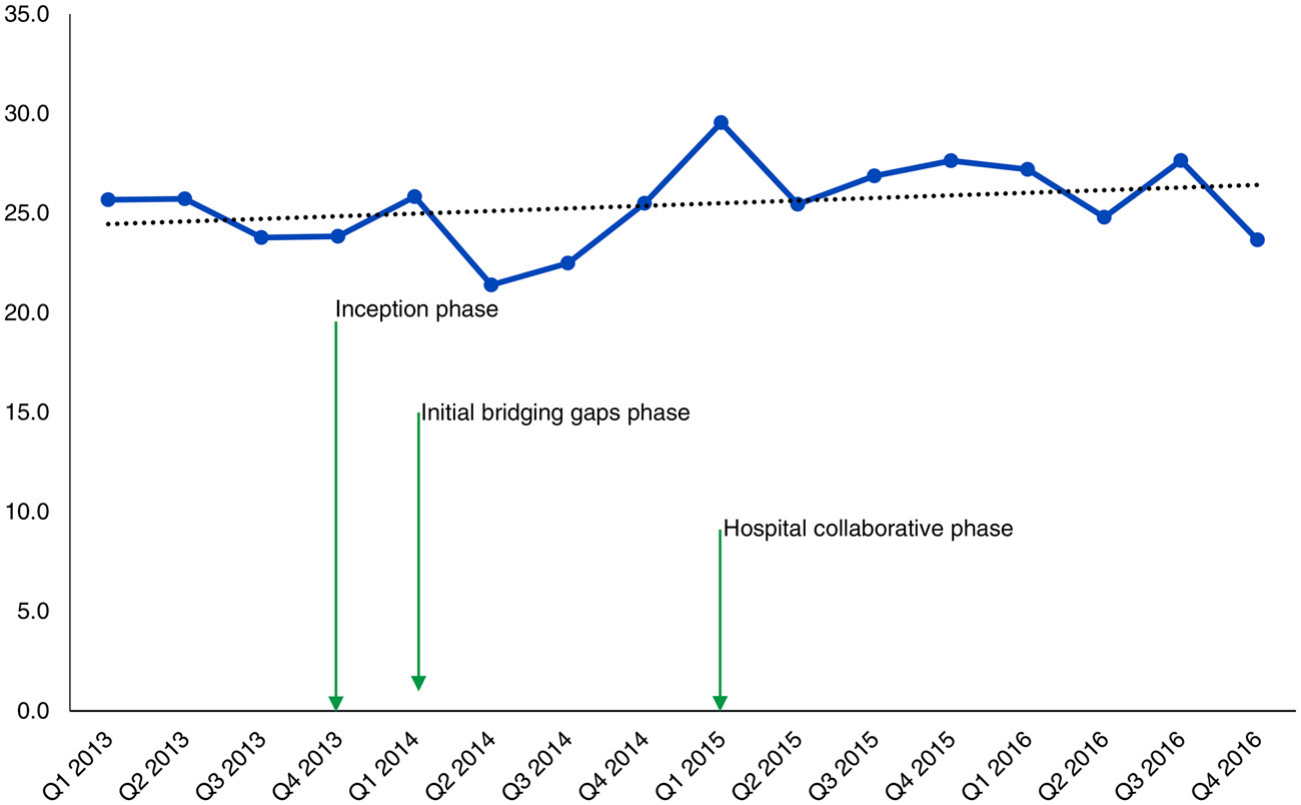
Cesarean Delivery Rate Trend Over Time in Hospitals Where a Locally Designed Package of Interventions to Improve Quality of Maternal and Newborn Care Was Implemented, Busoga Region, Uganda

In terms of impact on maternal and newborn outcomes, Figures 3 and 4 show a consistent decline in maternal and newborn deaths during the hospital collaborative phase. The decline occurred with minimal changes in the number of deliveries (Figure 5) and thus indicates improved outcomes. It is important to note that the first 2 quarters of the collaborative phase showed no change, perhaps because the actions had not yet reached saturation. As the phase progressed, deaths steadily declined. All the changes happened with minimal changes in the cesarean section rates (Figure 6), thus this impact is largely due to changes in basic obstetric care interventions.

We also witnessed a gradual rise in the number of deliveries. In the collaborative meetings, providers attributed the increases to quality improvement efforts as more people became aware of improved services. Uganda has an open referral system with no gatekeeping and with substantial bypassing, thus good quality might have driven demand.^15^ However, this hypothesis requires further analysis to confirm.

Lastly, among the achievements as shown in Table 4 after a single provision of catalytic supplies, hospitals were incentivized to sustain the provision of drugs, such as antenatal corticosteroids, phenobarbital for managing newborn convulsions, and magnesium sulfate for managing pre-eclampsia, as well as oxygen delivery systems and other management protocols.

**TABLE 4.** Baseline and Endline Assessment Results After Implementing Interventions to Improve Quality of Maternal and Newborn Care in 6 Hospitals, Busoga Region, Uganda

Readiness Assessment Items	December 2013 No. (%)	December 2016 No. (%)
Electricity supply on day of survey	6 (100)	6 (100)
Functional refrigerator	6 (100)	6 (100)
Motorized transport for referrals	5 (83)	6 (100)
Clean running water and soap	6 (100)	6 (100)
Functional newborn weighing scale	5 (83)	6 (100)
Thermometer	6 (100)	6 (100)
Pulse oximeter	4 (67)	6 (100)
Ambu-bag	6 (100)	6 (100)
Newborn suction device^a^	6 (100)	6 (100)
Oxygen (fully working delivery system)^a^	4 (67)	6 (100)
Nasogastric tubes and 20-ml syringes	4 (67)	6 (100)
Phototherapy machine^a^	2 (33)	6 (100)
Kangaroo Mother Care bed or chair^a^	1 (17)	6 (100)
IV cannular sets	6 (100)	6 (100)
IV bags and tubing	6 (100)	6 (100)
Feeding cups^a^	0 (0)	6 (100)
Fetoscope	6 (100)	6 (100)
Stethoscope	6 (100)	6 (100)
Clock^a^	4 (67)	6 (100)
Gloves (clean and sterile)	6 (100)	6 (100)
Adult weighing scale	6 (100)	6 (100)
**Drugs**		
Dextrose saline and rigors lactate	6 (100)	6 (100)
Vitamin K	6 (100)	6 (100)
Tetracycline eye ointment	6 (100)	6 (100)
Corticosteroids^a^	1 (17)	6 (100)
Oxytocin	6 (100)	6 (100)
Misoprostol	6 (100)	6 (100)
IV antibiotics (ceftriaxone, gentamicin, ampicillin)^a^	6 (100)	6 (100)
Local anesthetics (lidocaine)	6 (100)	6 (100)
Phenobarbital^a^	1 (17)	6 (100)
Nevirapine	6 (100)	6 (100)
Magnesium sulfate^a^	4 (67)	6 (100)
**Guidelines and protocols**		
Active management of the third stage of labor^a^	1 (17)	6 (100)
Management of postpartum hemorrhage^a^	1 (17)	6 (100)
Management of neonatal sepsis^a^	1 (17)	6 (100)
Newborn feeding^a^	1 (17)	6 (100)
Newborn resuscitation^a^	1 (17)	6 (100)

a Catalytic supplies provided by the project.

## REFLECTIONS AND IMPLICATIONS FOR PROGRAMS

We implemented a locally designed maternal and newborn quality improvement package in a regional network of hospitals. From this implementation, we achieved institutionalization of NCUs, kangaroo mother care spaces, resuscitation corners, and MPDRs in the maternity units. The package was designed and implemented in collaboration with local and national health system managers. We relied on available resources and deliberately implemented only “fit for context” interventions deemed affordable by the health system managers.

A key achievement of this work was the collaboration with facilities to set up infrastructures for infant care within the maternity units of all 6 hospitals, thus creating a regional network of care for mothers and newborns. In most general hospitals in Uganda, newborns are managed in the pediatric unit, which is typically located some distance away from the maternity unit.^12^ This is problematic for several reasons. First, precious time may be lost when transferring a newborn to the pediatric unit, which delays care and could be fatal. Second, the distance separates the mother, who remains in the maternity unit, from her baby, potentially hindering breastfeeding and bonding. Third, these transfers create extra work for already understaffed health providers. Locating the new NCUs within the maternity unit addresses all of these challenges.

A key achievement of this work was the collaboration with facilities to set up infrastructures for infant care within the maternity units of all 6 hospitals, thus creating a regional network of care for mothers and newborns.

Another important achievement of the project was enabling “action and response” from the MPDRs. Many countries report that the implementation of recommendations is a missing link in the MPDR process.^16^^,^^17^ Another key achievement was linking the MPDR to the plan-do-study-act cycle to help devise feasible solutions to address identified bottlenecks and solve the lack of action on MPDR recommendations. It is important to note that this time- and energy-consuming activity was challenging to conduct simultaneously with the MPDR meetings, especially at busy facilities. Nevertheless, we believe that this approach could solve the lack of action on MPDR recommendations. Second, through the engagement of leaders, the scope of the MPDR committee was broadened to give them more influence. For example, some facilities were able to access drugs from other facilities that did not use them.

Another key achievement was linking the MPDR to the plan-do-study-act cycle to help devise feasible solutions to address identified bottlenecks and solve the lack of action on MPDR recommendations.

Health system managers participated in the development, implementation, and evaluation of the project interventions, which offered 2 major benefits. First, the designed interventions were feasible within the context because managers generally knew what would or would not work in their context. Second, the managers endorsed the interventions and thus were more willing to cooperate with the implementation and maintenance of those things that were deemed useful. For example, facilities typically have an annual turnover of nurses from one department to another. This turnover was minimized because managers understood that after we built the capacity of staff in the NCUs, any transfers would lead to the loss of this competence.

In terms of resources, we mostly implemented interventions that could be achieved with the resources of the facility, in part because of limited funds for implementation but also because the health system managers tended to propose simple and affordable interventions that leveraged existing resources. This approach avoided the pitfalls of some past projects, which could not be sustained because they were too costly or conducted at a pace that the system could not maintain.^11^^,^^12^^,^^18^

The networking of facilities within the region also improved access to care by vulnerable families, as newborn care services were expanded from 1 health facility to 6. The project also helped free up the NCU at the regional referral hospital. Finally, the collaborative and mentorship program enabled health workers to consult each other easily and make better referrals.

## IMPLEMENTATION CHALLENGES

Although we targeted all health workers of maternity units for training and mentorships, most participants were nurses and midwives. Most medical doctors and clinical officers did not attend the training sessions and were rarely available for mentorships, mainly because they were busy with other work in the hospital or elsewhere, making them highly mobile and difficult to engage. This lack of engagement was a challenge because medical doctors are central in making care decisions for mothers and newborns. Future quality improvement and mentorship programs should be tailored to doctors' schedules to encourage their involvement and engagement. Where possible, hospital management and MOH staff should consider attaching medical doctors to only 1 or 2 departments where they hope to specialize, which would help build capacity in specialized areas instead of spreading resources across every department.

Future quality improvement and mentorship programs should be tailored to doctors' schedules to encourage their involvement and engagement.

Some of our change ideas failed. For example, 4 hospital teams proposed that all mothers caring for babies in the NCU should wear gowns to prevent infections. The hospital administration together with the project provided the resources for the gowns. However, this intervention did not work at any of the facilities. The gowns required regular washing and a clean place for changing, but there were no resources or mechanisms for either. Caregivers thus returned to wearing their own clothing. This idea needs further research to make it work in resource-limited hospitals. Another change idea with mixed results was having a medical doctor review all expectant mothers before birth. This idea was not practical in high-volume facilities with few doctors, but it worked well in low-volume facilities, especially the private not-for-profit hospitals.

We added some important medicines to the procurement lists of the hospitals, but occasional stock-outs still occurred, mostly due to an underestimation of supplies at the point of requisition at both the hospital and department levels and due to the general inadequacy of hospital budgets. At the department level, we encouraged the maternity nurse in charge to keep a buffer stock of vital supplies. However, the problem persists at the hospital and national medical stores level.

Another challenge with our approach was that it focused on improving service delivery through the lens of health workers and managers. There was limited engagement of end users or their representatives. As a consequence, we were not aware of the extent to which the package was patient-centered. Future implementations should prioritize and integrate end users' perspectives, as they are fundamental to improving the quality of care.

Other major challenges of this work included implementation in settings where staff were overwhelmed by the number of deliveries conducted and where habitual electricity outages affected operations at various units in the hospital, including the NCU and operating rooms. Moreover, although we were able to find spaces for the NCUs, they were not sufficient to meet demand. As a project, we were unable to address these persistent challenges, however, we acknowledge their existence and the dire need to address them.

## INTERVENTION INSTITUTIONALIZATION AND SUSTAINABILITY

This project became the basis for further improvement, sustainability, institutionalization, and scale-up. The project work led to an emergence of champions: the nurses and midwives, along with a few doctors, who are now passionate about newborn care and have become advocates and mobilizers for care and further improvement. The improvements we made have become a platform for continued internal and external work and investment. For instance, the platform was used by another quality improvement study, the Preterm Birth Initiative study, to test whether outcomes for preterm babies can be improved.^19^^,^^20^ Other projects, such as the Omwana Trial, also continue to expand infrastructure, staffing, and skills.^21^

As a team, in the second phase, we were able to move newborn care from hospitals to lower-level facilities by expanding the project to 6 high-volume primary health centers under the Maternal Newborn Scale Up Project (MANeSCALE). All of the health centers now have functional NCUs linked to and supported by the 6 hospitals.^22^ A film about the experiences and lessons learned is available at https://bit.ly/3tO6DYP.

The leadership engagement meetings led to the Busoga Maternal Newborn and Child health forum, a group of health actors who meet to discuss and promote issues about maternal, newborn, and child health in the region. Achievements of the forum include influencing MOH and Plan International to construct a bigger NCU at one of the hospitals,^23^ as well as the formation of a WhatsApp group in 2019 for leaders and health providers to discuss maternal and newborn health and other issues.^24^ These efforts indicate a continued expansion of the collaborative.

## CONCLUSION

This regionalized model of a hospital collaborative involving national and local leadership engagement, as well as the use of contextually appropriate interventions and available resources, presents an approach that can be applied by other quality improvement programs for designing feasible, sustainable, and scalable quality improvement interventions in resource-limited settings. A phased, capacity-building approach that empowers local health workers to become skilled and emerge as champions is critical for success. Our findings are important not only for Uganda's implementation of the Quality of Care Network but also for other countries in similar settings of high maternal and neonatal mortality.

## Notes

**Peer Reviewed**

**First published online:** May 5, 2021.
